# The memory engram: beginning the search

**DOI:** 10.1590/1980-5764-DN-2022-0059

**Published:** 2023-02-06

**Authors:** Eliasz Engelhardt, Gilberto Levy

**Affiliations:** 1 Universidade Federal do Rio de Janeiro, Instituto de Neurologia Deolindo Couto, Rio de Janeiro RJ, Brazil.; 2 Universidade Federal do Rio de Janeiro, Instituto de Psiquiatria, Rio de Janeiro RJ, Brazil.; 3 Independent Scholar, Rio de Janeiro RJ, Brazil.

**Keywords:** History, Memory, Memory Trace, Engram, História, Memória, Traço de Memória, Engrama

## Abstract

Some of the earliest conceptual milestones in memory research with relevance to the physical means through which its preservation is made possible, namely, the ‘memory trace’ or ‘engram’, are analysed in this study. The fundamental notions were laid down by Platon and Aristoteles. While Platon regarded memory as an imprint on a ‘wax block’ in the immortal soul, Aristoteles considered memory a modification in the mortal soul, imprinted like a cast at birth time. The Roman orators were interested in mnemotechnics, and Cicero is credited for the term ‘trace’ (*vestigium*) used for the first time. Much later, Descartes described the (memory) ‘trace’ (*trace*), linking psychic, and physical processes. Finally, Semon posited innovative concepts and terms centralized by the ‘engram’ (*Engramm*). The search of this important question, which begun about two and a half millennia ago, continues in focus, as can be seen through the growing rate of published papers on the subject.

## RESUMO

Alguns dos marcos conceituais iniciais na pesquisa de memória, com relevância para o meio físico, pelo qual a preservação desta é possibilitada, a saber, o ‘traço de memória’ ou ‘engrama’, são aqui analisados. As noções fundamentais foram formuladas por Platão e Aristóteles. Enquanto Platão via a memória como uma impressão em um ‘bloco de cera’ na alma imortal, Aristóteles considerava a memória uma modificação na alma mortal, impressa como um molde ao nascimento. Os oradores romanos tinham interesse em mnemotécnica e Cícero tem o crédito de ter usado o termo ‘traço’ (*vestigium*) pela primeira vez. Mais tarde, Descartes descreveu o ‘traço’ (*trace*) (de memória), ligando processos psíquicos e físicos. Finalmente, Semon propôs conceitos e termos inovadores centralizados pelo ‘engrama’ (*Engramm*). A busca dessa importante questão, que começou aproximadamente há dois milênios e meio, continua em foco, como pode ser visto pelo ritmo crescente de artigos publicados sobre o assunto.

## INTRODUCTION

Memory can be defined as the capacity of an organism to acquire and store information, ideas, or experiences at one time, maintaining them available for recollection (recall, recovery) at a subsequent time. The means through which preservation is made possible and provide a connection to the past by storing and somehow making available information about and from one's previous encounters is the ‘memory trace’ or ‘engram’^1,2^. In short, a memory trace is required to serve as the object of the remembering experience, a surrogate for the past event that is no longer available, and can be understood as an acknowledgement that memory has a physical basis, an organic substrate of the memory process, a structural analogue of the events it represents^2,3^.

The epoch when the interest on memory processes began to appear is not yet known, lost in the past. In this study, some of the early known milestones about the theme in Western civilization are discussed.

## GREEK SCHOLARS

**Figure 1:**
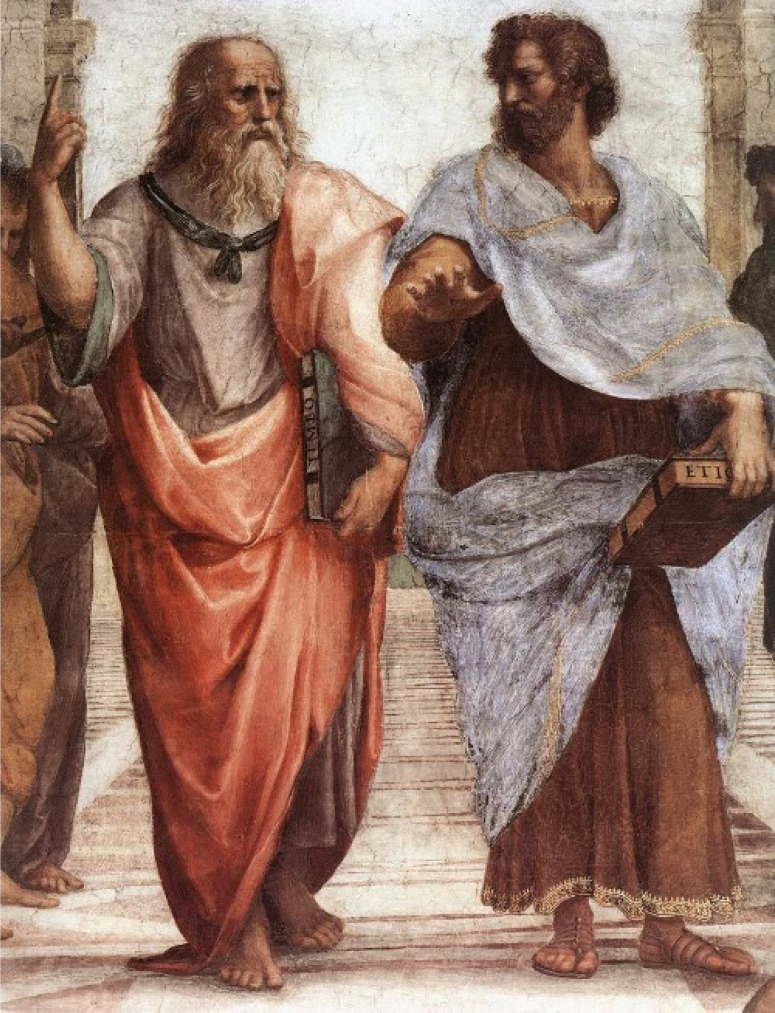
Plato (left) and Aristotle (right): a detail of ‘The School of Athens’ (1509–1511), a fresco by Raphael Sanzio, at the Raphael Rooms, Apostolic Palace, Vatican City. Public Domain.

Source: https://commons.wikimedia.org/wiki/File:Richard_Semon.jpg

The pivotal studies on memory really began with the notable Greek philosophers Platon and Aristoteles, who laid down the fundamental notions about the manner memory is acquired (and retrieved) (Figure 1). They were preceded by Socrates (ca 469–399 BCE), who, despite his acknowledged wisdom, made feel his influence mainly through the accounts of his disciples^4–6^.

Platon (427–347 BCE) apparently was the first one to develop a consistent approach to memory, discussed in many of his ‘Dialogues’, chronologically ordered in *Meno*, *Phaedo*, *Republic*, *Theaetetus*, *Philebus*, and *Timaeus*, as it is accepted^5,7^. Platon believed that the immortal soul (*psyche*), before the embodiment, has acquired complete knowledge of all ideas that make up the world during its travels (in *Meno*). However, when the soul is reborn (embodied), its knowledge is covert. But, through quest and study, what is already known [in latent form] can be remembered [recalled]. So, all knowledge is latent in the mind [since birth time], and never is forgotten. The process of recollection [*anamnesis*] merely elicits [not conscious] knowledge, raising it to consciousness^5,6,8,9^.

Later (in *Philebus*), it is affirmed that the soul takes awareness of the sensible things through the organs of senses, resulting in ‘preservation of perception’^10^. Such contradiction is explored (in *Phaedo*): “… if it is true…that our learning is nothing else than recollection, then this would be an additional argument that we must necessarily have learned in some previous time what we now remember…”^5,11^. So, a positive role to ‘perception’ in the acquisition of knowledge is given, i.e., one cannot attain knowledge without perception^5^.

The discussion about how and where lies the memory is considered through two metaphors — of the ‘wax tablet’ and the ‘aviary’.

The ‘wax tablet metaphor’ (in *Theaetetus*) suggests that the memory is imprinted (stamped) as with a ‘sealing ring’ on a ‘wax block’ [wax tablet] (a gift of *Mnemosyne* — Greek divinity of memory), located in the soul^4,12^.

The ‘aviary metaphor’ was proposed (also in *Theaetetus*) as an alternative to the former. There, birds are captured and gathered in an ‘aviary’ (birdcage), each representing different kinds of knowledge [memories]. The aviary is empty [blank] in the newborn, and any bird that is captured and encaged corresponds to a learned topic [knowledge] [memory]^4,12^. This metaphor is in contradiction of Platon's initial idea of a pre-existent knowledge. However, it is useful to explain the inquiry (in *Phaedo*) on how memory is acquired by the immortal soul.

Both metaphors, in different ways, refer implicitly to the concept of a ‘memory trace’ – in the first already imprinted in the soul (*psyche*) [mind], and in the second, acquired (learned) later.

Aristoteles (384–322 BCE), philosopher and biologist, was a disciple of Platon. He also focused on memory, mainly in his ‘On Memory and Recollection’ (*De Memoria et Reminiscentia*), regarded as the first scientific study on memory, and also in his ‘On the Soul’ (*De Anima*)^13^. He received some concepts from his mentor and introduced many new ones, some contradictory to those of his precursor^3,4,13^.

Aristoteles believes that the soul (*psyche*) is mortal, including the part called ‘mind’ (*nous*) [intellect], contradicting Platon. Therefore, a newborn comes with a soul [mind] without previous knowledge (impassive, blank), but potentially capable to acquire knowledge, comparable to a ‘blank’ or ‘erased writing-tablet’ [as a ‘wax-tablet’] in condition to receive some written information [‘blank tablet (*tabula rasa*) concept’], based on learning and experience^6,13^.

Aristoteles explains: “…Memory is neither perception nor conceptual thought, but some permanent condition or modification of one of these, dependent upon lapse of time…”. And further: “…the modification arising from sense-perception in the sentient soul and in the part of the body where sense resides, as if it were a picture of the real thing, and memory we call the permanent existence of this modification…”. Then: “…the stimulus involved in the act of perception imprints as it were a mould of the sense-affection [impression of the percept] exactly as a seal-ring acts in stamping…[‘memory trace’]”^14^. Thus, “…memory is the permanence of an image [*phantasmatos*] [‘memory trace’] regarded as the copy (image) [*eikonos*] of the object it images [*phantasma*]…”^14^.

After Platon and Aristoteles, an array of philosophers, physicians, theologists who continued the studies and writings about memory can be cited, and among these, Zenon, Posidonius, Seneca, Plotinos, Galenus, Origenes, and others, covering a period of about a half millennium, who offered varied concepts, some endorsing those of Platon, others those of Aristoteles, but without introducing new concepts on the subject^3,4,15^.

The ‘memory trace’ concept, up to this time, appeared only in a covert way, i.e., implicit to the proposed concepts. Next, the (memory) ‘trace’ makes its entrance in an explicit manner.

## ROMAN ORATORS – THE ‘TRACE’

Some orators, at the turn of the millennium, emphasized the importance of memory in rhetoric^3,4,16^.

Marcus Tullius Cicero (1^st^ century BCE), Roman statesman, lawyer, philosopher, and orator, in his ‘Tusculan Disputations’ (*Tusculanarum Disputationum*), ponders about the soul and the nature of memory: “Shall we suppose that the soul receives impressions like on wax, and that memory consists of ‘vestiges’ (*vestigia*) [traces, marks] of the things sealed in the mind?”. Thus, for the first time, the term ‘trace’ [‘memory trace’] appeared^16–18^.

The educator and orator Fabius Marcus Quintilianus (ca 35-ca 100 CE), in his ‘Institutes of Oratory’ (*Institutio Oratoria*) (ca 95 CE) deal with the theory and practice of rhetoric, and emphasizes the requirement of a good memory for this skill^16^.

Overall, the Roman orators contributed very scarcely to explain memory mechanisms, as their concern was mainly about mnemotechnics.

## MEDIEVAL PERSONAGES

The Middle Ages or Medieval Period revealed philosophers and other scholars who examined memory questions. As examples, can be cited Augustine of Hippo, Nemesius of Emesa, Albertus Magnus, and mostly Thomas Aquinas, spanning almost one millennium, without providing new concepts, as the main focus continued to be on mnemotechnics^3,16,19^.

## THE RENAISSANCE AND DESCARTES

The more representative authors of the Renaissance who dealt with memory were Descartes, who deserves major admiration, and Malebranche.

René Descartes (1596–1650) was a French mathematician, physicist, and philosopher. In his ‘The Man’ (*L'Homme*) (1664) he explains memory formation based on the peculiar brain structure he proposed, formed by small filaments and tubules through which the ‘animal spirit’ flows, according to the movements of the pineal gland. The stimuli from the real object that strike the small filaments of the organs of the senses cause the opening of adequate small tubules, the entering of the ‘animal spirit’, and the outline of a figure (image) in the interior surface of the brain, related to the real object. Such state improves progressively, permitting certain tubules to remain open, even after the action of the real object has ceased. This is the reason why these images do not erase easily, but are retained there, permitting that the ideas be formed there again after a long time, without requiring the presence of the objects to which they relate. However, if they close again, at least they leave ‘traces’ (*traces*) [‘memory traces’] in this part of the brain, so that they can reopen more easily in the same way, under the influence of the pineal gland^20–23^. The term ‘trace’ (*trace* in French), was translated as *vestigia* in the Latin editions of ‘The Man’, with the meaning of ‘memory trace’^22^.

Nicolas Malebranche (1638–1715), French priest and philosopher, published his ‘The Search After Truth’ (*Recherche de la Vérité*) (1674–1675) following the ideas of Descartes, without providing new contributions to the subject^4,24^.

## MODERN PERIOD

The Modern Period revealed some personalities, who proposed varied kinds of hypothesis to explain memory mechanisms. Among them should be cited mainly Thomas Hobbes, John Locke, David Hartley, Charles Bonnet, and Jules Bernard Luys, covering a period of about three centuries, who presented views that were soon discarded^3,16,19^.

**Figure 2:**
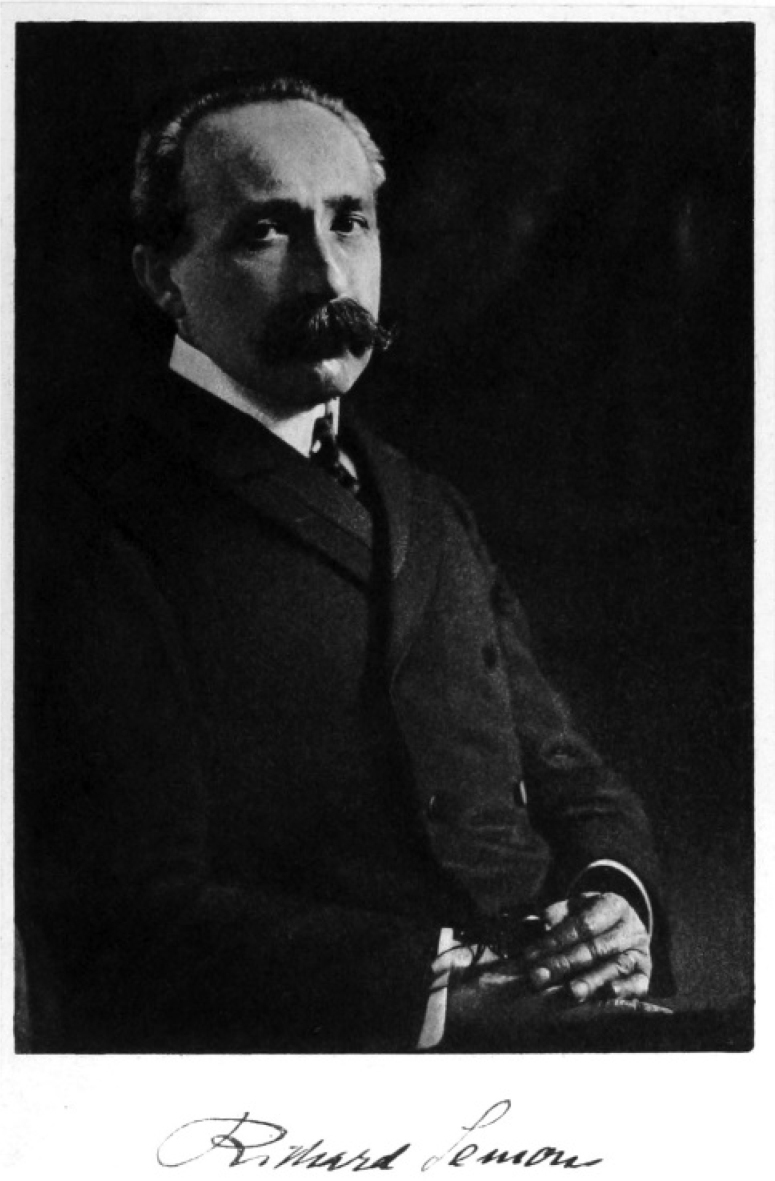
Richard Semon (anonymous/unknown author) (before 1918).

Source: https://commons.wikimedia.org/wiki/File:Richard_Semon.jpg

And finally appeared Semon (Figure 2).

Richard Wolfgang Semon (1859–1918), German physician and biologist, focused on memory research and published two books about the theme, ‘The Mneme’ (*Die Mneme*) (1904) and the ‘Mnemic Psychology’ (*Die mnemischen Empfindungen*) (1909)^25,26^. There, Semon explains: “When an organism has been temporarily stimulated…after the cessation of the stimulus…it can be shown that such organism…has been permanently affected. I call this action of the stimulus as its ‘engraphic action’ (*engraphische Wirkung*) [encoding] because a permanent record has been engraved or inscribed (*eingräbt oder einschreibt*) on the ‘organic substance’ [nervous tissue]”. Then, “I designated as the ‘engram’ (*Engramm*) of the given stimulus such permanent change of the ‘organic substance’ (*organische Substanz*) [‘memory trace’] …”^25^. And concludes, “The phenomena resulting from the existence of one or more engrams in an organism I describe as ‘mnemic phenomena’ [memory]”^25^. The engram can be recovered (evoked, recalled) through ‘ecphory’ (*Ekphorie*), which corresponds to the “…passage of an engram from a latent to a manifest state…”^25–27^. He expanded his theory defining more concepts, as the ‘engram complex’ (“…the stimulus…is invariably of a complex nature…[this corresponds to]…simultaneous excitation-complex…generate a simultaneous engram-complex”), ‘engram-store’ (*Engrammschatz*) (the sum of the engrams held by an organism), and many others^26^.

Semon introduced new concepts and terms to explain the mechanism of memory, the basic ones being ‘engraphy’ [encoding], ‘engram’ [‘memory trace’], and ‘ecphory’ [recall], and expanded these concepts with others, of a more complex nature^25,27^. Lamentably, his work was ignored for a long time, allegedly due to his support of the thesis that memory has a hereditary mechanism, a Lamarckian view that was not accepted by the scientific community at the time^27^.

## COMMENTS

**Figure 3:**
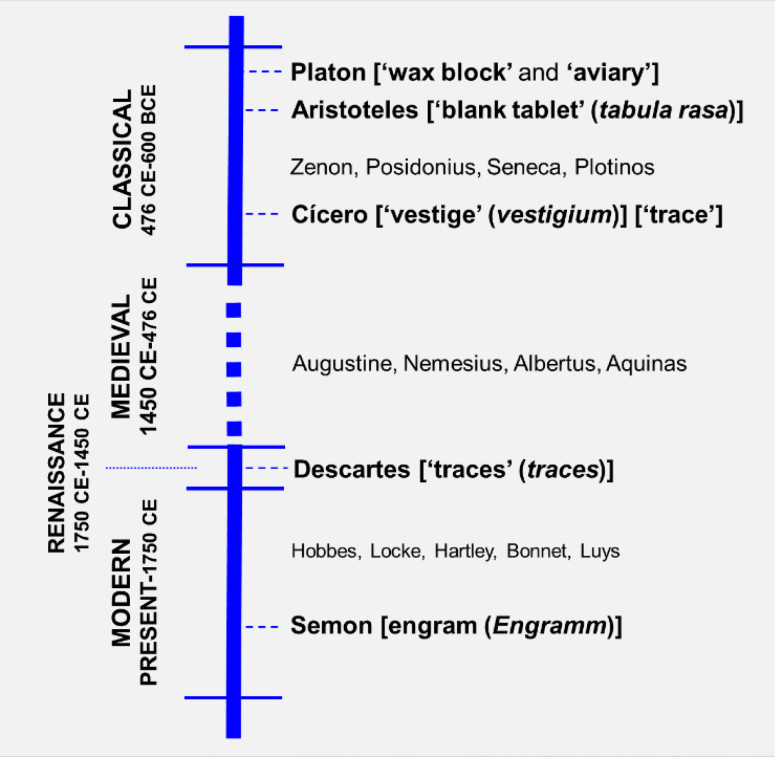
Timeline of the main ‘trace’ studies. Left side: time periods, right side: scholars and their proposals for memory mechanisms.

Source: https://examples.yourdictionary.com/historicaleras-list-of-major-time-periods-in-histori.html

The ‘memory trace’ appears in nearly every account of memory and emerges in an implicit manner in the works of Platon and of Aristoteles, who laid down the foundations for memory studies. Platon, who believed in an immortal soul, regarded memory as an imprint on a ‘wax block’ (inborn knowledge, brought with the embodied soul), or ‘birds’ in an aviary (acquired knowledge after birth), as expressed in his metaphors. Aristoteles, who believed in a mortal soul, considered memory a permanent condition or modification in the sentient soul, imprinted like a cast, similarly to a sealring used in stamping, beginning at birth time. They were followed by numerous philosophers, physicians, and other thinkers, who failed to provide any new contribution to the subject. The Roman orators, more interested in mnemotechnics, contributed poorly. However, Cicero is credited with using the term ‘trace’ (*vestigium*) (‘memory trace’) for the first time, but without giving further details. Much later appeared Descartes, providing a new concept, linking psychic and physical processes, and defining the (memory) ‘trace’. Finally came Semon, postulating innovative concepts and terms centralized by the ‘engram’ concept [‘memory trace’] and accompanied by ‘engraphy’ [encoding] and ‘ecphory’ [recall], and others more^25–27^, as can be seen in the timeline graphic (Figure 3). Although innovative, it is possible to identify in Semon's ideas many of those formulated by the ancient predecessors. His concepts, ignored for a long time, were revived by Karl Lashley, as seen in his paper ‘In search of the engram’^28^. Although Lashley concluded that it was not possible to localize an engram, his work yielded important information for many further studies^29^. The search of this important question, which begun about two and a half millennia ago, continues in focus and advancing in large steps, as can be seen through the growing rate of published papers on the subject.
